# Ethno-Racial Differences in Age and Symptom Severity Among Pre-Menopausal Women Commencing Treatment for Benign Gynecological Conditions with a Levonorgestrel-Releasing Intrauterine Device

**DOI:** 10.1089/heq.2024.0238

**Published:** 2025-06-11

**Authors:** Michael J. Green, Kemi M. Doll, Mollie E. Wood, Annie G. Howard, Lauren G. Anderson, Joacy G. Mathias, Natalie A. Rivadeneira, Erin T. Carey, Timothy S. Carey, Wanda Nicholson, Til Stürmer, Evan R. Myers, Whitney R. Robinson

**Affiliations:** ^1^ Department of Obstetrics and Gynecology, Duke University School of Medicine, Durham, North Carolina, USA.; ^2^ Department of Obstetrics and Gynecology, University of Washington School of Medicine, Seattle, Washington, USA.; ^3^ Department of Epidemiology, Gillings School of Global Public Health, University of North Carolina at Chapel Hill, Chapel Hill, North Carolina, USA.; ^4^ Department of Epidemiology and Department of Biostatistics, Gillings School of Global Public Health, University of North Carolina at Chapel Hill, Chapel Hill, North Carolina, USA.; ^5^ Department of Obstetrics and Gynecology, School of Medicine, University of North Carolina at Chapel Hill, Chapel Hill, North Carolina, USA.; ^6^ Cecil G Sheps Center for Health Services Research, University of North Carolina at Chapel Hill, Chapel Hill, North Carolina, USA.; ^7^ Milken Institute School of Public Health, George Washington University, Washington, DC, USA.; ^8^ Department of Obstetrics and Gynecology and Margolis Center for Health Policy, Duke University School of Medicine, Durham, North Carolina, USA.

**Keywords:** racial groups, ethnicity, intrauterine devices, uterine diseases, uterine bleeding, pelvic pain

## Abstract

**Introduction:**

Levonorgestrel-releasing intrauterine devices (LNG-IUDs) can be effective treatments for benign gynecological conditions, but there may be ethno-racial differences in how patients receive treatment.

**Methods:**

Data were from a health care system in the U.S. South (April 2014–September 2019). We identified 783 female patients aged 18–44 years with an LNG-IUD for a benign gynecological condition (455 White, 208 Black, and 120 Hispanic patients). Abstraction of medical notes preceding insertion gave symptom severity scores for uterine bleeding, pelvic pain, and uterine bulk. Linear and negative binomial regression models assessed differences in patients’ age and symptom severity scores, respectively. Covariates included insurance status, parity, prior treatments, and fibroid and endometriosis diagnoses.

**Results:**

White patients’ mean age was 32.4 years. Black patients were similarly aged (+0.9 years [95% confidence interval: −0.4 to 2.1]), whereas Hispanic patients were older (+3.4 years [2.0–4.9]), and adjustment attenuated this difference (+0.7 [−0.7 to 2.0]). Estimated ratios indicated more severe bleeding and bulk symptoms for Black and Hispanic than White patients (bleeding: Black: 1.7[1.5–2.0], Hispanic: 1.7[1.4–2.1]; bulk: Black: 1.5[1.3–1.9], Hispanic: 1.5[1.2–1.9]). Adjustment for covariates attenuated estimates, especially for Hispanic patients (bleeding: Black: 1.4[1.2–1.6], Hispanic: 1.2[1.0–1.4]; bulk: Black: 1.3[1.1–1.6], Hispanic: 1.2[1.0–1.6]).

**Discussion:**

At the time of LNG-IUD insertion, Hispanic patients were older than White patients. Black and Hispanic patients had more severe symptoms than White patients. Differences in age and symptom severity were associated with lack of insurance coverage, higher parity, presence of fibroids, and prior medical management, potentially indicating barriers to early LNG-IUD treatment for Black and Hispanic patients.

## Introduction

Abnormal uterine bleeding (AUB), that is, bleeding with irregular frequency, duration, or volume,^1^ is common and often debilitating. Prevalence estimates for women of reproductive age range between 10% and 30%,^2–4^ and AUB often goes unreported.^1,2,5–7^ AUB is associated with poorer physical and mental well-being,^3^ reduced quality of life, and billions of dollars of estimated economic costs (direct costs of medical treatment and indirect via lost productivity).^2^ Uterine bleeding can also indicate benign gynecological conditions such as endometriosis or uterine fibroids.^8,9^

Levonorgestrel-releasing intrauterine devices (LNG-IUDs) are recommended as early treatment for benign gynecological conditions (and are approved for such use by the U.S. Food and Drug Administration),^8,10–12^ but may be underutilized relative to invasive surgical treatments such as hysterectomy.^11,13–15^ LNG-IUDs effectively control bleeding and pain symptoms^8,16–18^ and are more cost-effective for patients than hysterectomy, with lower risk of adverse events^19–21^ and no extended recovery period preventing economic activity.^7^ LNG-IUDs may be less effective for patients with fibroids, who experience higher rates of expulsion.^8,18^ Patients might prefer hysterectomy for definitive and permanent symptom control.^21–23^ Patients may also prefer avoiding hormonal medications, particularly via inserted devices requiring further medical intervention to remove or maintain. Understanding how patients present for LNG-IUD treatment may help identify barriers to use, including differences by ethno-racial group.

Complex, historical, and sociocultural processes have marginalized particular ethno-racial categories while privileging others.^24^ The “weathering” hypothesis suggests that cumulative social and economic disadvantages (i.e., structural racism), interpersonal racism, discrimination, and associated stressors may lead marginalized groups, over the life course, to earlier onset of unfavorable physical health conditions,^25,26^ including gynecological problems.^27^ Black women experience higher prevalence, severity, and earlier onset for uterine fibroids than White women,^28–32^ and incidence is associated with lifetime experiences of racial discrimination.^31^ Weathering is most often applied to explaining poorer health among Black populations, but generalizes to Hispanic populations.^26^ Women from marginalized groups may need gynecological treatment earlier and have more severe symptoms.

Marginalized ethno-racial groups may also experience disadvantages in treatment access.^27,33^ Disparities in medical insurance can mean being treated by providers with fewer resources and less training.^34^ For example, Medicaid patients are less likely to receive minimally invasive than abdominal hysterectomy surgery.^35^ Furthermore, distrust of the medical system (based on historical injustices or patients’ own or friends’ and families’ experiences of discrimination) or concerns regarding costs can lead to delaying treatment.^7,22,36,37^ Structural barriers to LNG-IUD treatment are poorly understood, but women from marginalized ethno-racial groups use contraceptives less frequently,^33,38^ including IUDs.^39^ It is unclear whether the causes of lower contraceptive use generalize to gynecological LNG-IUD treatment.

Patients from marginalized ethno-racial groups may experience more severe gynecological symptoms and more barriers to treatment. We, therefore, addressed the following research questions with data on pre-menopausal women receiving LNG-IUD treatment for a benign gynecological condition:

1. Are there ethno-racial differences in the age at which patients commence LNG-IUD treatment?
2. Are there ethno-racial differences in the severity of gynecologic symptoms preceding LNG-IUD treatment?
3. To what extent are any ethno-racial differences in age of treatment and symptom severity associated with differences in patient characteristics such as insurance, parity, prior treatment, and diagnostic categories?

## Methods

### Sample

LNG-IUD patients were identified in structured electronic health records (EHRs) from a large hospital system in the U.S. South. The hospital system comprises a large academic center with large and small community hospitals, has an extensive charity care program, and covers metropolitan, suburban, and rural areas. Unstructured EHR notes and other medical records from the 4 months before LNG-IUD insertion were manually abstracted to obtain data on clinical history using a defined protocol^40–42^ and matched with structured data warehouse records. Eligible women were aged 18–44 years, with an LNG-IUD related medical encounter between April 4, 2014, and September 18, 2019, and an associated diagnostic billing code for a benign gynecological condition (*n* = 1,081; benign gynecological indication defined as follows: AUB, fibroids, endometriosis, or gynecological pain; see Supplementary Table S1 for the ICD and CPT codes). Patients receiving the LNG-IUD for contraceptive purposes without a diagnostic code for a benign gynecological condition were ineligible (emphasizing specificity for gynecological treatment over sensitivity). Each patient’s first LNG-IUD encounter within the study period was treated as the index insertion. The study was approved by the University of North Carolina Institutional Review Board (17-2728). The hospital system was not within a Medicaid expansion state during this period.

**FIG. 1.**
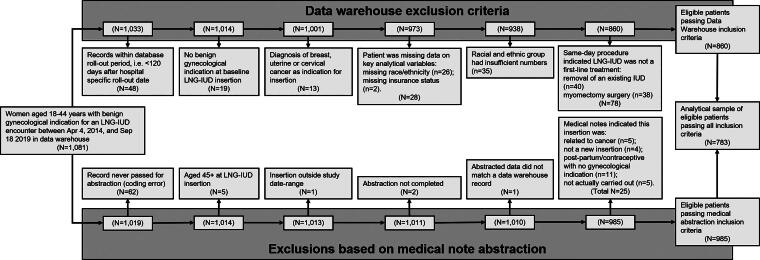
Analytical sample inclusion flowchart. Exclusion criteria were related to: (1) the structured data warehouse records (*left-hand side* of the diagram); and (2) the abstraction of unstructured surgical notes and medical records (*right-hand side* of the diagram). Patients were excluded if structured data warehouse records indicated: <120 days of pre-insertion medical records to establish baseline symptoms (due to database rollout timing; *n* = 48); no benign gynecological indication associated with the baseline LNG-IUD insertion (i.e., gynecological indications were only present for post-baseline LNG-IUD related encounters; *n* = 19); a cancer indication for the LNG-IUD insertion (*n* = 13; see Supplementary Table S1 for ICD codes); patients were missing data on key analytical variables (*n* = 28); patients identified with an ethno-racial group with insufficient numbers to provide stable estimates and avoid identifiability (*n* = 35, including non-Hispanic patients identifying as: American Indian, Alaska Native, Asian, Native Hawaiian, Pacific Islander, or Mixed Race; descriptive data are included in Supplementary Table S2); or a same-day procedure indicated that an LNG-IUD may have already been in use (same-day removal of an existing LNG-IUD, *n* = 40) or was being used post-operatively (with myomectomy, *n* = 38). Patients were also excluded if abstraction of medical records had not taken place (due to a coding error; *n* = 62), was incomplete (*n* = 2), could not be matched to data warehouse records (*n* = 1), or if abstraction indicated the insertion: took place at age 45 years or more (*n* = 5); was outside the study date range (*n* = 1); was related to cancer (*n* = 5); was not a new insertion (*n* = 4); was for postpartum/contraceptive purposes with no gynecological indication (*n* = 11); or was not actually carried out (*n* = 5). LNG-IUD: levonorgestrel-releasing intrauterine device.

Aiming to identify new episodes of treatment for a benign gynecological condition with an LNG-IUD (not necessarily patients’ first treatment), we excluded patients if there was evidence that the LNG-IUD treatment: was for cancer; was not for a benign gynecological condition; was supporting another procedure (myomectomy); or if data were incomplete or out-of-scope (Fig. 1). Patients were also excluded if there was an indication that an LNG-IUD was already present, either in the clinical notes or if there was a same-day LNG-IUD removal. Following 298 exclusions, our analytical sample comprised 783 patients passing all exclusion criteria. Since this represented all the available data, no *post hoc* power analyses were conducted.^43^

### Measures

Sociodemographic data, including baseline age, race, ethnicity, and insurance status, were available as structured data in the EHR extraction. Age in years was centered on its mean value (33.1 years). Patients reported race and ethnicity at registration and were coded as any of the following: non-Hispanic Black, Hispanic, non-Hispanic White (hereafter: Black, Hispanic, and White). Patients identifying as non-Hispanic and either American Indian, Alaska Native, Asian, Native Hawaiian, or Pacific Islander or belonging to multiple non-White races were excluded because combining heterogeneous cultural and ethnic identities into a single group is uninformative (especially so if the group is small) and no single group contained enough patients to avoid risk of identifiability (*n* = 35 exclusions across all racial groups). Insurance status categories were as follows: commercial or employment-linked insurance (hereafter referred to as “commercial”; including Blue Cross Blue Shield, Commercial, State Health Plan, or Tricare), Medicaid (or other government agency), Uninsured (“self-pay” assumed to be uninsured), or Medicare (including Medicare advantage).

Medical abstraction of unstructured data provided clinical information on parity, prior treatment, and gynecological symptom severity. Abstraction was based on physicians’ notes over the 4 months preceding LNG-IUD insertion (including notes from the insertion itself). In those notes, clinicians often documented earlier clinical and treatment history from across the life course.

Parity (based on number of prior deliveries) was coded as follows: not recorded, nulliparous, single parity, or multiparous. Three measures of prior treatment were derived, organized around medical management, surgical procedures, and IUD use. Prior medical treatments were categorized as none, single type, or multiple types based on the presence of the following treatments: Depo Provera, gonadotropin-releasing hormone (GnRH) agonists, hormonal contraceptives (oral, patch, or ring), intravenous hormonal injections, hormonal implants, iron supplements, tranexamic acids (i.e., Lysteda), or progestins. Similarly, prior surgical procedures were categorized as none, single type, or multiple types based on history of the following: dilation and curettage, endometrial ablation, hysteroscopy, laparoscopy (for gynecological indication), laparotomy (for gynecological indication), or myomectomy. Prior IUD treatment was coded as follows: no prior use, prior use of an IUD (type unknown), or prior use of a hormonal IUD. Structured data also indicated if diagnostic codes for fibroids and endometriosis were associated with the LNG-IUD insertion.

Gynecological symptom severity was coded using adapted versions of symptom severity indices developed for hysterectomy patients.^44^ The original symptom indices used data, which were available for our LNG-IUD patients (unstructured physician notes, administrative billing codes) and data that were unavailable (structured pre-operative physician notes, laboratory and pathology test results, and pharmacy billing data). Supplementary Table S2 details the adaptation of the indices to the more limited data available for LNG-IUD patients (necessarily relying heavily on physicians’ documentation). Three distinct scales represented the following: the severity of uterine bleeding symptoms (range: 0–21; median: 4), the severity of pelvic pain symptoms (range: 0–16; median: 3), and the severity of uterine bulk symptoms (range: 0–7; median: 1), with higher scores indicating more severe symptoms. Uterine bleeding and pelvic pain severity scores are interpreted as indicative for LNG-IUD treatment, and the uterine bulk severity score as relatively contraindicative for LNG-IUD use (since fibroids are associated with expulsion).^8,18^

### Analyses

Analyses were performed in SAS system, version 9.4.^45^ We first present descriptive statistics, both overall and by race and ethnicity, with appropriate statistical tests for differences between groups (chi-square tests for categorical variables and *t*-tests for continuous variables).

Average differences in mean age between ethno-racial groups were estimated using linear regression models (outcome: age; ethno-racial group: predictor). Following unadjusted models, we adjusted separately for insurance status, parity, prior treatments (medical, surgical, and IUDs), and diagnostic categories (fibroids and endometriosis). A final model included all covariates.

Ethno-racial differences in symptom severity were modeled with negative binomial models, as symptom severity scores were skewed toward zero (see Supplementary Fig. S1). Separate models were estimated for bleeding, pain, and bulk. An estimated ratio of 1.5 for Black patients means that Black patients’ scores were 1.5 times higher on average than scores in the reference group (White patients). Models were separately adjusted for the following: age at insertion, insurance status, parity, prior treatments (medical, surgical, and IUDs), and diagnostic categories (fibroids and endometriosis); with a final model including all covariates.

Covariate adjustments were not intended as adjustment for confounders (since none are likely causes of race and ethnicity) nor was estimating main effects of covariates our aim. Rather, we aimed to compare associations with ethno-racial group before and after adjustment to gain insights into factors associated with ethno-racial differences in modeled outcomes. For example, attenuation of associations between ethno-racial group and symptom severity under adjustment for insurance indicates that ethno-racial differences in symptom severity were associated with ethno-racial differences in insurance.

## Results

Mean age at treatment for White patients was 32.4 years and was similar for Black patients (33.2 years), whereas Hispanic patients were older (35.8 years; Table 1). Compared with White patients, Black and Hispanic patients had more severe uterine bleeding and bulk symptom scores, were more likely to have AUB and fibroid codes, and less likely to have gynecological pain, endometriosis, or multiple gynecological codes than White patients. Hispanic patients were considerably more likely to be uninsured than White patients (58% vs. 11%), while Black patients were more often insured via Medicaid than White patients (32% vs. 15%). Compared with White patients, Black patients were more likely to be primiparous, while Hispanic patients were more likely to be multiparous. Black and Hispanic patients more commonly had multiple types of prior medical treatment and were less likely to have had a prior surgical procedure than White patients. There were no clear ethno-racial differences in prior IUD experience.

**Table 1.** Characteristics of Levonorgestrel-Releasing Intrauterine Device Patients in a Large U.S. South Health Care System from 2014 to 2019

	White	Black	Hispanic	*p*-Value for difference by ethno-racial group	All patients
Total *N*	455	208	120		783
					**Mean (SD)**
Age	32.4 (7.6)	33.2 (7.1)	35.8 (6.9)	<0.001	33.1 (7.4)
Uterine bleeding symptom severity score	3.8 (3.8)	6.4 (5.3)	6.5 (5.3)	<0.001	4.9 (4.7)
Pelvic pain symptom severity score	3.5 (3.3)	3.2 (3.2)	3.1 (3.0)	0.358	3.3 (3.2)
Uterine bulk symptom severity score	1.3 (1.7)	2.0 (1.9)	2.0 (1.9)	<0.001	1.6 (1.8)
					***N* (%)**
Diagnostic billing codes associated with the LNG-IUD insertion					
Abnormal uterine bleeding	299 (65.7)	165 (79.3)	98 (81.7)	<0.001	562 (71.8)
Gynecological pain	237 (52.1)	64 (30.8)	30 (25.0)	<0.001	331 (42.3)
Endometriosis	86 (18.9)	25 (12.0)	<10^a^	0.003	120 (15.3)
Fibroids	31 (6.8)	34 (16.4)	18 (15.0)	<0.001	83 (10.6)
Number of above diagnostic categories associated with the LNG-IUD insertion					
1 only	289 (63.5)	147 (70.7)	93 (77.5)	0.008	529 (67.6)
2 or more	166 (36.5)	61 (29.3)	27 (22.5)	0.008	254 (32.4)
Insurance status					
Commercial	325 (71.4)	103 (49.5)	34 (28.3)	<0.001	462 (59.0)
Medicaid	69 (15.2)	66 (31.7)	15 (12.5)	<0.001	150 (19.2)
Uninsured	48 (10.5)	24 (11.5)	70 (58.3)	<0.001	142 (18.1)
Medicare	13 (2.9)	15 (7.2)	<10^a^	0.004	29 (3.7)
Parity					
Not recorded	26 (5.7)	16 (7.7)	13 (10.8)	0.135	55 (7.0)
Nulliparous	191 (42.0)	74 (35.6)	26 (21.7)	<0.001	291 (37.2)
Single parity	65 (14.3)	43 (20.7)	11 (9.2)	0.014	119 (15.2)
Multiparous	173 (38.0)	75 (36.1)	70 (58.3)	<0.001	318 (40.6)
Prior medical treatments^b^					
None	152 (33.4)	51 (24.5)	31 (25.8)	0.039	234 (29.9)
Single type	182 (40.0)	69 (33.2)	47 (39.2)	0.235	298 (38.1)
Multiple types	121 (26.6)	88 (42.3)	42 (35.0)	<0.001	251 (32.1)
Prior surgical procedures^c^					
None	254 (55.8)	130 (62.5)	76 (63.3)	0.146	460 (58.8)
Single type	133 (29.2)	43 (20.7)	21 (20.7)	0.007	197 (25.2)
Multiple types	68 (15.0)	35 (16.8)	23 (19.2)	0.505	126 (16.1)
Any prior IUD use					
None recorded	357 (78.5)	170 (81.7)	99 (82.5)	0.466	626 (80.0)
Prior IUD (uncertain type)	32 (7.0)	<10^a^	<10^a^	0.360	50 (6.4)
Prior IUD (hormonal)	66 (14.5)	29 (13.9)	12 (10.0)	0.438	107 (13.7)

^a^ Cell sizes <10 and their associated percentages are suppressed.

^b^ Prior medical treatments included: Depo Provera, GnRH agonists, hormonal contraceptives (oral, patch, or ring), intravenous hormonal injections, hormonal implants, iron supplements, Lysteda (tranexamic acids), or progestins.

^c^ Surgical procedures included: dilation and curettage, endometrial ablation, hysteroscopy, laparoscopy (for gynecological indication), laparotomy (for gynecological indication), and myomectomy.

LNG-IUD, levonorgestrel-releasing intrauterine device.

Linear regression models estimated that Hispanic patients were 3.4 years older than White patients, whereas Black patients were treated at similar ages to White patients (Table 2). Age differences between White and Hispanic patients were attenuated after adjustment for insurance status, parity, or diagnostic indications, while adjustment for prior treatments did not appreciably change the estimates. After adjusting for all factors, there was no strong evidence for residual age differences between White and Hispanic patients.

**Table 2.** Estimated Differences in Average Age at Levonorgestrel-Releasing Intrauterine Device Treatment by Ethno-Racial Group in a Large U.S. South Health Care System from 2014 to 2019** **

Difference in age (compared with the reference group) estimated from linear regression models for age at LNG-IUD treatment (95% CIs)						
	Unadjusted	Adjustment for insurance status	Adjustment for parity	Adjustment for prior treatments	Adjustment for diagnostic categories	Adjustment with all covariates
Black (ref: White)	0.85 (−0.35 to 2.05)	0.74 (−0.47 to 1.95)	0.57 (−0.47 to 1.60)	0.84 (−0.36 to 2.04)	0.34 (−0.86 to 1.53)	0.28 (−0.76 to 1.31)
Hispanic (ref: White)	3.43 (1.95 to 4.90)	2.17 (0.55 to 3.78)	1.77 (0.48 to 3.06)	3.37 (1.91 to 4.83)	2.81 (1.35 to 4.27)	0.68 (−0.68 to 2.04)
Medicaid (ref: Commercial)		−0.83 (−2.19 to 0.52)				−2.48 (−3.63 to −1.34)
Uninsured (ref: Commercial)		2.80 (1.29 to 4.31)				1.50 (0.23 to 2.77)
Medicare (ref: Commercial)		5.04 (2.31 to 7.76)				4.61 (2.33 to 6.89)
Single Parity (ref: Nulliparous)			5.80 (4.45 to 7.15)			5.82 (4.53 to 7.11)
Multiparous (ref: Nulliparous)			8.59 (7.57 to 9.60)			8.48 (7.49 to 9.48)
Unknown parity (ref: Nulliparous)			4.14 (2.31 to 5.96)			4.09 (2.36 to 5.84)
Prior medical treatments^a^—single type (ref: none)				−1.24 (−2.49 to 0.00)		−0.86 (−1.89 to 0.17)
Prior medical treatments^a^—multiple types (ref: none)				−1.00 (−2.32 to 0.33)		−0.20 (−1.30 to 0.90)
Prior surgical procedures^b^—single type (ref: none)				−1.38 (−2.60 to −0.16)		−0.80 (−1.90 to 0.30)
Prior surgical procedures^b^—multiple types (ref: none)				1.74 (0.30 to 3.19)		1.11 (−0.11 to 2.34)
Some prior IUD treatment—type unknown (ref: none)				2.11 (0.03 to 4.20)		0.18 (−1.56 to 1.92)
Prior treatment with a hormonal IUD (ref: none)				2.48 (0.99 to 3.97)		2.05 (0.81 to 3.29)
Diagnostic code for fibroids (ref: not present)					3.06 (1.41 to 4.71)	3.22 (1.82 to 4.61)
Diagnostic code for endometriosis (ref: not present)					−3.22 (−4.63 to −1.82)	−1.10 (−2.41 to 0.21)

^a^ Prior medical treatments included: Depo Provera, GnRH agonists, hormonal contraceptives (oral, patch, or ring), intravenous hormonal injections, hormonal implants, iron supplements, tranexamic acids (Lysteda), or progestins.

^b^ Surgical procedures included: dilation and curettage, endometrial ablation, hysteroscopy, laparoscopy (for gynecological indication), laparotomy (for gynecological indication), and myomectomy.

CI, confidence interval; LNG-IUD, Levonorgestrel-releasing intrauterine device.

The mean bleeding severity score was 3.8 for patients who were White. Negative binomial models indicated that Black and Hispanic patients had bleeding scores that were 1.7 times higher on average than those of White patients (Table 3). Differences were particularly attenuated with adjustment for insurance (especially for Hispanic patients) and prior treatment.

**Table 3.** Estimated Ratios Between Levonorgestrel-Releasing Intrauterine Device Patients’ Baseline Uterine Bleeding Symptom Severity Scores by Ethno-Racial Group in a Large U.S. South Health Care System from 2014 to 2019

Estimated ratios (compared with reference groups)^a^ for uterine bleeding severity scores (95% CIs)							
	Unadjusted	Adjusted for age	Adjustment for insurance status	Adjustment for parity	Adjustment for prior treatment	Adjustment for diagnostic categories	Adjustment with all covariates
Black (ref: White)	1.70 (1.46–1.98)	1.70 (1.46–1.97)	1.63 (1.39–1.90)	1.69 (1.47–1.97)	1.45 (1.27–1.67)	1.67 (1.43–1.95)	1.36 (1.18–1.56)
Hispanic (ref: White)	1.72 (1.43–2.08)	1.60 (1.33–1.93)	1.44 (1.17–1.77)	1.62 (1.34–1.97)	1.51 (1.28–1.78)	1.67 (1.39–2.02)	1.18 (0.99–1.42)
Age at LNG-IUD treatment (ref: mean age)		1.02 (1.01–1.03)					1.02 (1.01–1.03)
Medicaid (ref: Commercial)			1.21 (1.02–1.44)				1.18 (1.00–1.37)
Uninsured (ref: Commercial)			1.48 (1.22–1.79)				1.38 (1.17–1.62)
Medicare (ref: Commercial)			1.30 (0.92–1.83)				1.26 (0.93–1.71)
Single Parity (ref: Nulliparous)				1.17 (0.95–1.43)			1.09 (0.91–1.32)
Multiparous (ref: Nulliparous)				1.31 (1.12–1.53)			1.14 (0.97–1.33)
Unknown parity (ref: Nulliparous)				1.22 (0.93–1.60)			1.13 (0.89–0.143)
Prior medical treatments^b^—single type (ref: none)					1.64 (1.41–1.92)		1.70 (1.47–1.98)
Prior medical treatments^b^—multiple types (ref: none)					2.89 (2.47–3.39)		3.04 (2.61–3.54)
Prior surgical procedures^c^—single type (ref: none)					0.93 (0.81–1.08)		1.02 (0.88–1.19)
Prior surgical procedures^c^—multiple types (ref: none)					1.32 (1.12–1.55)		1.30 (1.11–1.52)
Some prior IUD treatment—type unknown (ref: none)					1.08 (0.85–1.38)		0.99 (0.78–1.26)
Prior treatment with a hormonal IUD (ref: none)					0.87 (0.73–1.04)		0.86 (0.72–1.02)
Diagnostic code for fibroids (ref: not present)						1.06 (0.85–1.31)	0.99 (0.82–1.20)
Diagnostic code for endometriosis (ref: not present)						0.77 (0.64–0.93)	0.77 (0.64–0.93)

^a^ Estimated with negative binomial models, which offered superior model fit to Poisson models (likelihood ratio; *p* < 0.01), and the ratio of expected to observed zero scores (1.02) did not strongly indicate a need for a zero-inflated model.

^b^ Prior medical treatments included: Depo Provera, GnRH agonists, hormonal contraceptives (oral, patch, or ring), intravenous hormonal injections, hormonal implants, iron supplements, tranexamic acids (Lysteda), or progestins.

^c^ Surgical procedures included: dilation and curettage, endometrial ablation, hysteroscopy, laparoscopy (for gynecological indication), laparotomy (for gynecological indication), and myomectomy.

CI, confidence interval; LNG-IUD, Levonorgestrel-releasing intrauterine device.

There was little evidence of ethno-racial disparities in severity of pelvic pain symptoms (Supplementary Table S3). Covariate adjustments did not considerably impact the estimated ratios for pain severity.

Black and Hispanic patients’ bulk symptom severity scores were approximately 1.5 times greater than for White patients (Table 4). Differences between Black and White patients were mildly attenuated with adjustment for age and insurance, but most sensitive to adjustment for fibroids. Differences between Hispanic and White patients were most sensitive to adjustment but also attenuated with adjustment for age, insurance, parity, and fibroids.

**Table 4.** Estimated Ratios Between Levonorgestrel-Releasing Intrauterine Device Patients’ Baseline Uterine Bulk Symptom Severity Scores by Ethno-Racial Group in a Large U.S. South Health Care System from 2014 to 2019

Estimated ratios (compared with reference groups)^a^ for uterine bulk severity scores (95% CIs)							
	Unadjusted	Adjusted for age	Adjustment for insurance status	Adjustment for parity	Adjustment for prior treatment	Adjustment for diagnostic categories	Adjustment with all covariates
Black (ref: White)	1.52 (1.25–1.86)	1.47 (1.22–1.77)	1.46 (1.19–1.78)	1.52 (1.25–1.84)	1.50 (1.23–1.83)	1.43 (1.17–1.74)	1.32 (1.10–1.60)
Hispanic (ref: White)	1.51 (1.19–1.93)	1.25 (0.99–1.57)	1.44 (1.10–1.89)	1.40 (1.11–1.78)	1.48 (1.16–1.88)	1.45 (1.14–1.84)	1.24 (0.97–1.58)
Age at LNG-IUD treatment (ref: mean age)		1.06 (1.05–1.07)					1.05 (1.04–1.06)
Medicaid (ref: Commercial)			1.33 (1.06–1.66)				1.43 (1.16–1.77)
Uninsured (ref: Commercial)			1.14 (0.89–1.48)				0.97 (0.77–1.23)
Medicare (ref: Commercial)			1.00 (0.63–1.60)				0.89 (0.58–1.37)
Single Parity (ref: Nulliparous)				1.63 (1.26–2.11)			1.34 (1.04–1.72)
Multiparous (ref: Nulliparous)				1.92 (1.58–2.33)			1.40 (1.13–1.74)
Unknown parity (ref: Nulliparous)				1.16 (0.80–1.66)			0.93 (0.65–1.33)
Prior medical treatments^b^—single type (ref: none)					0.79 (0.64–0.98)		0.81 (0.66–0.99)
Prior medical treatments^b^—multiple types (ref: none)					1.10 (0.89–1.37)		1.14 (0.94–1.40)
Prior surgical procedures^c^—single type (ref: none)					1.09 (0.88–1.34)		1.13 (0.92–1.39)
Prior surgical procedures^c^—multiple types (ref: none)					1.38 (1.09–1.74)		1.28 (1.03–1.59)
Some prior IUD treatment—type unknown (ref: none)					1.17 (0.83–1.65)		1.10 (0.80–1.51)
Prior treatment with a hormonal IUD (ref: none)					1.11 (0.87–1.43)		0.98 (0.78–1.24)
Diagnostic code for fibroids (ref: not present)						1.71 (1.32–2.21)	1.60 (1.26–2.03)
Diagnostic code for endometriosis (ref: not present)						0.94 (0.74–1.21)	1.19 (0.93–1.53)

^a^ Estimated with negative binomial models, which offered superior model fit to Poisson models (likelihood ratio; *p* < 0.01), and the ratio of expected to observed zero scores (0.94) did not strongly indicate a need for a zero-inflated model.

^b^ Prior medical treatments included: Depo Provera, GnRH agonists, hormonal contraceptives (oral, patch, or ring), intravenous hormonal injections, hormonal implants, iron supplements, tranexamic acids (Lysteda), or progestins.

^c^ Surgical procedures included: dilation and curettage, endometrial ablation, hysteroscopy, laparoscopy (for gynecological indication), laparotomy (for gynecological indication), and myomectomy.

CI, confidence interval; LNG-IUD, levonorgestrel-releasing intrauterine device.

Patients who had been excluded from the analysis because they received the LNG-IUD alongside a surgical myomectomy procedure (*n* = 38) were older, had more severe uterine bleeding and bulk symptoms, had more prior treatments, and were more likely to be Black than White (Supplementary Table S4). Patients excluded because they received the LNG-IUD the same day that another LNG-IUD was removed (*n* = 40) had less severe symptoms and fewer prior treatments but did not differ by ethno-racial group. Repeating regression models with these 78 patients included yielded findings consistent with the main analyses (Supplementary Table S5). Regression models were also repeated with only those patients who had diagnostic codes for fibroids (Supplementary Table S6) or endometriosis (Supplementary Table S7). There was not strong evidence for differences in age or symptom severity among the 83 patients with fibroid diagnoses but confidence intervals were wide. Among the 120 patients with endometriosis, Hispanic patients were still older than White patients and had more severe bulk symptoms (with the latter difference being robust to adjustment). Black endometriosis patients had more severe bleeding symptoms than their White counterparts.

## Discussion

Black and Hispanic compared with White LNG-IUD patients in the U.S. South had more severe uterine bleeding and bulk symptoms documented in their EHRs in the months before LNG-IUD insertion. In addition, Hispanic LNG-IUD patients were older than White patients. Attenuation of differences in adjusted models indicated that ethno-racial differences in patients’ insurance, childbearing patterns, treatment history, and fibroids prevalence may help explain some of the observed age and symptom differences among LNG-IUD patients.

Existing evidence on ethno-racial differences in access to and effectiveness of LNG-IUDs comes from the contraceptive literature.^39^ Limited gynecological literature indicates that LNG-IUDs may be underutilized as early treatment for benign gynecological conditions among U.S. Black and Hispanic patients.^11,13–15^ For instance, observational studies of fibroid patients^28,32^ and retrospective studies of hysterectomy patients^15,29^ indicate more severe gynecological symptoms for Black and Hispanic than for White patients, with little evidence that more alternative treatments are attempted before hysterectomy.^15^ However, Black and Hispanic gynecological patients may prefer or have greater access to non-IUD treatments: in our study, they were more likely than White patients to have attempted multiple prior medical treatments before an LNG-IUD, but less likely to have had prior gynecological surgery.

More severe symptoms for Black than White patients could represent some combination of developing more severe disease earlier in life (e.g., because of “weathering”^25,26^), not receiving treatment until symptoms have progressed in severity (i.e., disparities in access), or disfavoring LNG-IUDs and postponing their use until preferred treatments have failed to manage symptoms. Consistent with the weathering hypothesis, Black women develop fibroids at earlier ages than White women,^28,29,32,46^ and our findings indicated that higher fibroid prevalence may partially explain Black LNG-IUD patients’ more severe bulk symptoms. Qualitative studies indicate that Black women may delay treatment due to concerns regarding finances and insurance coverage,^7,22^ but we only found mild attenuation of differences in symptom severity between Black and White patients with adjustment for insurance status. Black–White differences in bleeding severity were more attenuated after accounting for Black patients receiving multiple prior medical treatments. This may reflect both the severity of bleeding among Black patients and greater resistance to LNG-IUD use (as documented in the contraceptive literature) versus other treatments. Furthermore, considerable differences in symptom severity remained for Black patients, even after adjustment for these factors. This implies other mechanisms of disadvantage, potentially including stresses associated with perceived discrimination,^7,31^ or differing cultural norms regarding treatment seeking, perhaps as a result of historic or contemporary structural racism.^7,24^ For example, Black women are more likely than White women to believe the government uses contraception to limit minority populations,^47^ so may be less likely to choose long-acting treatments that are commonly used for contraception.

Data on gynecological treatment and symptom severity are sparse for Hispanic women.^30,48^ We found Hispanic LNG-IUD patients approximately 3 years older when commencing treatment than White patients. Hispanic patients’ older age at treatment was attenuated after accounting for their greater likelihood of being uninsured (58% vs. 11% among Black and White patients) and multiparous (58% vs. 38%). Regarding symptom severity, Hispanic patients had more severe bleeding and bulk symptoms than White patients, consistent with findings from one population survey.^49^ Hispanic-White differences in severity of bleeding were most attenuated with adjustment for insurance and treatment history, while differences in bulk symptoms were most attenuated with adjustment for age at treatment and parity. As with Black patients, Hispanic patients may not receive LNG-IUD treatment until symptoms are more severe because of disparities in access to early LNG-IUD treatment (e.g., lack of insurance coverage was especially common for Hispanic patients) or preferences for trying other medical treatments before LNG-IUDs. In addition, our results indicate that family building may contraindicate the use of LNG-IUDs, which are contraceptives in addition to being gynecological treatments.

Alternatively, treatment may not be delayed *per se*. Hispanic patients may simply not develop their more severe symptoms until older ages, but it is not clear why such a pattern would be associated with insurance, parity, or prior treatment. Approximately 3 in 4 Hispanic patients had documentation indicating prior attempts at medical management. We lack details on dates or duration, but the high prevalence of previous treatment suggests that symptoms had often been present for some time among Hispanic patients before LNG-IUD treatment.

Findings were from a large, not-for-profit hospital system in the U.S. South and may not generalize to other geographic areas or kinds of health systems. Limiting our sample to patients with diagnostic codes for benign gynecological conditions may have missed patients receiving an LNG-IUD for both contraception and treatment of a benign condition, who were only being coded for the primary contraceptive purpose, and ethno-racial or insurance-related differences in application of coding are plausible. We also focused on patients receiving LNG-IUD treatment without capturing patients who went untreated or chose alternative treatments, introducing the possibility of selection bias. Given that Black and Hispanic patients had tended to try multiple alternatives before LNG-IUDs, differences from White patients may reflect differences in health care delivery, patient preferences, and LNG-IUD access in addition to differences in gynecological severity. Relatedly, LNG-IUDs may not be optimal for patients with large fibroids,^8,18^ and Black and Hispanic patients were more likely to have fibroid diagnoses and were overrepresented among LNG-IUD patients who were excluded for receiving the LNG-IUD post-surgically with a myomectomy (a treatment for fibroids). Encouragingly, findings were similar when these surgical patients were included.

Abstraction of records in the months preceding LNG-IUD treatment allowed more detailed measurement of symptom severity than in claims-based or large-scale survey studies.^44^ Despite that strength, a limitation of an EHR-based study is reliance on physician documentation instead of patient-reported symptoms. There are known racial biases toward under-ascertainment of pain for Black patients,^50^ potentially explaining why pain symptoms showed no ethno-racial disparities and diverged from other studies showing more severe pain for Black patients.^28,32^ Information on duration of symptoms was also limited,^41^ which restricted our ability to distinguish delayed treatment from older ages of symptom onset.

## Health Equity Implications

At LNG-IUD placement, Hispanic patients were older than White patients, and a greater likelihood of being uninsured, multiparous, and having fibroids appeared to account for some of this difference. Compared with White patients, Black and Hispanic patients had more often tried multiple alternative medical treatments first and had more severe uterine bleeding and bulk symptoms at insertion. Ethno-racial differences in age, insurance, parity, treatment history, and the presence of fibroids were associated with differences in symptom severity. Findings suggest differentially lower utilization of LNG-IUDs as early treatments for benign gynecological conditions. Future research should address potential barriers to accessing LNG-IUD treatment for Black and Hispanic patients, such as lack of insurance coverage, conflicts with family building, and other reasons why Black and Hispanic patients may disfavor LNG-IUDs as gynecological treatments.

## Data Sharing Statement

The data analyzed in this study are owned and administered by the North Carolina Translational and Clinical Sciences Institute (NC TRACS). Access to the data for research purposes can be requested at: https://tracs.unc.edu/index.php. Questions about data access may be directed to: nctracs@unc.edu.

## Abbreviations Used

AUB: Abnormal uterine bleeding
CI: Confidence interval
CPT: Current procedural terminology
EHR: Electronic health records
GnRH: Gonadotropin-releasing hormone
ICD: International classification of diseases
IUD: Intrauterine device
LNG-IUD: Levonorgestrel-releasing intrauterine device
SAS: Statistical Analysis System
SD: Standard deviation
