# Supplementary material for: Ethno-Racial Differences in Age and Symptom Severity Among Pre-Menopausal Women Commencing Treatment for Benign Gynecological Conditions with a Levonorgestrel-Releasing Intrauterine Device
Source: Health Equity. 2025 Jun 11;9(1):326–38. doi: 10.1089/heq.2024.0238 (PMC12241843; doi:10.1089/heq.2024.0238)
Supplement: Supplementary Data [file heq.2024.0238_supplementary_data.docx]

**Supplementary Material**

Contents:

1. Supplementary Table S1: Medical Codes Utilized
2. Supplementary Table S2. Mapping of Existing Composite Symptom Severity Index for Bulk, Vaginal Bleeding, and Pelvic Pain onto Available Data for IUD patients
3. Supplementary Figure S1: Distribution box-plots of symptom severity scores among LNG-IUD patients
4. Supplementary Table S3: Estimated ratios between LNG-IUD patients’ baseline pelvic pain symptom severity scores by ethno-racial group in a large US South Healthcare system from 2014-2019
5. Supplementary Table S4: Comparison of analysis sample to excluded LNG-IUD patients
6. Supplementary Table S5: Sensitivity analyses with an extra 78 LNG-IUD patients who were excluded from the main analyses
7. Supplementary Table S6: Sensitivity analyses with 83 LNG-IUD patients who had diagnostic codes for Fibroids
8. Supplementary Table S7: Sensitivity analyses with 120 LNG-IUD patients who had diagnostic codes for Endometriosis

**Supplementary Table S1: Medical Codes Utilized**

| **Diagnostic Category** | **ICD 9 Codes** | **ICD 10 Codes** | **CPT Codes** |
| --- | --- | --- | --- |
| Abnormal Uterine Bleeding | 626.x | N91.x, N92.x, N93.x |  |
| Fibroids | 211.8, 215.6, 218.x, 219.x | D20.0, D20.1, D21.5, D25.x, D26.x, D27.x, D28.x |  |
| Endometriosis | 617.x | N80.x |  |
| Gynecologic Pain | 338.x, 625.x, 789.x | G89.x, N94.x, R10.x |  |
| Uterine Bulk or Pelvic Mass | 620.x, 621.2, 789.3 | N83.x, N85.2 |  |
| Anemia | 280.x, 285.x | D50.x, D62.x, D63.x, D64.9 |  |
| Cancer | 153.x, 154.x, 158.x, 159.x, 173.5, 174.x, 179.x, 180.x, 181.x, 182.x, 183.x, 184.x, 185.x, 186.x, 187.x, 188.x, 189.x, 195.3, 197.6, 198.x | C18.x, C20.x, C21.x, C50.x, C51.x, C52.x, C53.x, C54.x, C55.x, C56.x, C57.x, C58.x, C67.x, C68.x, C76.3, C79.6, C79.82, C79.81 |  |
| LNG-IUD |  |  | 58300^a^, S4989^a^, S4981 |

.x indicates that all sub-categories were included.

^a^These CPT codes do not specifically indicate Levonorgestrel IUDs, so additional searches were made on the patient’s ordered medications for the terms “intrauterine” and “Levonorgestrel”.

**Supplementary Table S2. Mapping of Existing Composite Symptom Severity Index for Bulk, Uterine Bleeding, and Pelvic Pain onto Available Data for LNG-IUD patients**

|  | **Symptom severity score indices developed with a sample of hysterectomy patients^a^** | **Adapted symptom severity score indices using available data for IUD patients** |
| --- | --- | --- |
| **Bulk Severity Index**  **(potential range: 0-13)** | | |
| **Points** | **Symptoms** | |
| 1 | Bloating (PT) | Bloating (PT) |
|  | Pelvic Pressure (PT) | Pelvic Pressure (PT) |
|  | Uterine Size 50^th^-75^th^ Percentile (LAB) | Largest numerical record of uterine size from ultrasound, MRI, CT scan, or pelvic exam in 50^th^-75^th^ percentile, i.e. 8-9cm (PT) |
| 2 | Bulk Diagnosis Code at Surgery (DX) | - |
| 3 | Non-specified Bulk Symptoms (PT) | Non-specified Bulk Symptoms (PT) |
|  | Bulk as Indication for Surgery (MD) | - |
|  | Bulk Diagnosis Code in Year Prior to Surgery (DX) | Bulk or Pelvic Mass Diagnosis Code at Insertion (DX) |
| 4 | Uterine Size ≥ 75^th^ Percentile (LAB) | Largest numerical record of uterine size from ultrasound, MRI, CT scan, or pelvic exam in ≥75^th^ percentile, i.e. ≥9cm, or uterus was described as “enlarged” (PT) |
| **Uterine Bleeding Severity Index**  **(potential range: 0-28)** | | |
| **Points** | **Symptoms** | |
| 1 | Vaginal Bleeding Diagnosis Code at Surgery (DX) | - |
|  | Heavy Bleeding (PT) | Heavy Bleeding (PT) |
|  | Irregular Bleeding (PT) | Irregular Bleeding (PT) |
|  | Heavy Bleeding as Indication for Surgery (MD) | - |
| 2 | Irregular Bleeding as Indication for Surgery (MD) | - |
|  | Vaginal Bleeding Diagnosis Code in Year Prior to Surgery (DX) | Vaginal Bleeding Diagnosis Code at Insertion (DX) |
|  | Period Last Longer than 7 Days (PT) | Period Last Longer than 7 Days (PT) |
|  | Lethargia or Dizziness (PT) | Lethargia or Dizziness (PT) |
| 3 | Iron Use (MD) | Iron Use (PT) |
|  | ER Visit Related to Bleeding (DX) | - |
|  | Anemia Diagnosis Code at Surgery (DX) | Anemia (PT) |
| 4 | Anemia - HGB < 10 (LAB) | - |
|  | Anemia as Indication for Surgery (MD) | - |
|  | More than 1 ER Visit Related to Bleeding (DX) | At least 1 ER visit for gynecologic symptoms (PT) |
|  | ER Visit Related to Anemia (DX) | - |
|  | Anemia Diagnosis Code in Year Prior to Surgery (DX) | - |
| 5 | History of Blood Transfusion (MD) | Had a blood transfusion (PT) |
|  | More than 1 ER Visit Related to Anemia (DX) | Anemia Diagnosis Code at Insertion (DX)^b^ |
| **Pain Severity Index**  **(potential range: 0-20)** | | |
| **Points** | **Symptoms** | |
| 1 | Pelvic Pain (PT) | Pelvic Pain (PT) |
|  | Painful Periods (PT) | Painful Periods (PT) |
|  | Painful Intercourse (PT) | Painful Intercourse (PT) |
|  | Tylenol (PHARM) | Tylenol (PT) |
|  | - | NSAID (PT)^c^ |
| 2 | NSAID (PHARM) | - |
|  | Pain as Indication for Surgery (MD) | - |
|  | Painful Periods as Indication for Surgery (MD) | - |
| 3 | Pain Diagnosis Code In Year Prior to Surgery (DX) | - |
|  | Pain Diagnosis Code at Surgery (DX) | - |
|  | Other Pain Medication (PHARM) | Other Pain Medication (PT) |
| 4 | Opioid (PHARM) | Opiates (PT) |
|  | At Least 1 Pain Related ER Visit (DX) | At least 1 ER visit for gynecologic symptoms (PT) |
|  | Muscle Relaxant (PHARM) | - |
|  | - | Pain Diagnosis Code at Insertion (DX)^b^ |

**^a^**See Doll *et al.* Development of an algorithm to assess unmeasured symptom severity in gynecologic care. *Am J Obstet Gynecol.* 2022 Mar;226(3):388.e1-388.e11. Scores calculated by summing the points allocated to each item present.

^b^These items were assigned higher scores because there was only a single diagnosis code item vs multiple items in the version developed with hysterectomy patients.

^c^This item was down-graded because of the difference in source (pharmacy billing vs physician notes) and was therefore considered to be more likely to represent over the counter medications.

Key: PT: Patient reported symptom or patient information as recorded in unstructured physician notes; DX: Administrative billing code (ICD9, ICD10, CPT); MD: Physician indicated reason for procedure in preoperative or operative notes (unavailable for IUD patients); LAB: Results from laboratory tests or pathology report (unavailable for IUD patients); PHARM: Prescription information from pharmacy billing data (unavailable for IUD patients).

**
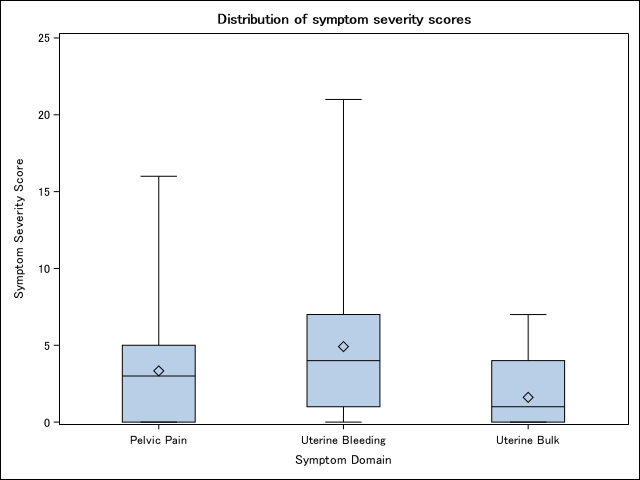
**

**Supplementary Figure S1. Distribution box-plots** **of symptom severity scores among LNG-IUD patients**

**Supplementary Table** **3: Estimated ratios between LNG-IUD patients’ baseline pelvic pain symptom severity scores by ethno-racial group in a large US South Healthcare system from 2014-2019**

| **Estimated ratios (compared to reference groups)^a^ for pelvic pain severity scores**  **(95% CIs)** | | | | | | | |
| --- | --- | --- | --- | --- | --- | --- | --- |
|  | **Unadjusted** | **Adjusted for age** | **Adjustment for insurance status** | **Adjustment for parity** | **Adjustment for prior treatment** | **Adjustment for diagnostic categories** | **Adjustment with all covariates** |
| Black (ref: White) | 0.91  (0.76 to 1.08) | 0.92  (0.77 to 1.10) | 0.87  (0.73 to 1.05) | 0.92  (0.77 to 1.10) | 0.87  (0.74 to 1.04) | 0.97  (0.82 to 1.16) | 0.85  (0.71 to 1.01) |
| Hispanic (ref: White) | 0.90  (0.72 to 1.12) | 0.98  (0.78 to 1.23) | 0.80  (0.62 to 1.02) | 0.99  (0.79 to 1.24) | 0.92  (0.74 to 1.13) | 1.03  (0.83 to 1.27) | 0.88  (0.70 to 1.10) |
| Age at LNG-IUD treatment (ref: mean age) |  | 0.98  (0.97 to 0.99) |  |  |  |  | 1.00  (0.98 to 1.01) |
| Medicaid (ref: Commercial) |  |  | 1.13  (0.92 to 1.38) |  |  |  | 1.38  (1.14 to 1.67) |
| Uninsured (ref: Commercial) |  |  | 1.30  (1.04 to 1.62) |  |  |  | 1.43  (1.16 to 1.76) |
| Medicare (ref: Commercial) |  |  | 1.06  (0.70 to 1.60) |  |  |  | 1.33  (0.91 to 1.95) |
| Single Parity (ref: Nulliparous) |  |  |  | 0.81  (0.64 to 1.02) |  |  | 0.89  (0.71 to 1.11) |
| Multiparous (ref: Nulliparous) |  |  |  | 0.74  (0.62 to 0.88) |  |  | 0.88  (0.73 to 1.06) |
| Unknown parity (ref: Nulliparous) |  |  |  | 0.64  (0.46 to 0.88) |  |  | 0.75  (0.56 to 1.01) |
| Prior medical treatments^b^ – single type  (ref: none) |  |  |  |  | 1.34  (1.12 to 1.61) |  | 1.31  (1.10 to 1.56) |
| Prior medical treatments^b^ – multiple types  (ref: none) |  |  |  |  | 1.93  (1.61 to 2.33) |  | 1.90  (1.59 to 2.28) |
| Prior surgical procedures^c^ – single type  (ref: none) |  |  |  |  | 1.68  (1.42 to 1.99) |  | 1.33  (1.11 to 1.58) |
| Prior surgical procedures^c^ – multiple types (ref: none) |  |  |  |  | 1.43  (1.17 to 1.74) |  | 1.27  (1.05 to 1.55) |
| Some prior IUD treatment -type unknown  (ref: none) |  |  |  |  | 0.94  (0.70 to 1.26) |  | 1.06  (0.80 to 1.41) |
| Prior treatment with a hormonal IUD  (ref: none) |  |  |  |  | 0.88  (0.71 to 1.09) |  | 0.88  (0.72 to 1.08) |
| Diagnostic code for fibroids  (ref: not present) |  |  |  |  |  | 0.97  (0.76 to 1.23) | 0.98  (0.77 to 1.24) |
| Diagnostic code for endometriosis  (ref: not present) |  |  |  |  |  | 2.09  (1.73 to 2.54) | 1.80  (1.48 to 2.20) |

^a^Estimated with negative binomial models, which offered superior model fit to Poisson models (likelihood ratio; p<0.01), and the ratio of expected to observed zero scores (0.83) did not strongly indicate a need for a zero-inflated model.

^b^Prior medical treatments included: Depo Provera, GnRH agonists, hormonal contraceptives (oral, patch, or ring), intravenous hormonal injections, hormonal implants, iron supplements, tranexamic acids (Lysteda), or Progestins.

^c^Surgical procedures included: dilation and curettage, endometrial ablation, hysteroscopy, laparoscopy (for gynecologic indication), laparotomy (for gynecologic indication), and myomectomy.

**Supplementary Table S4: Comparison of analysis sample to excluded LNG-IUD patients**

|  |  |  |  | **P-value for difference** | **All Patients** |
| --- | --- | --- | --- | --- | --- |
|  | **Analysis sample** | **Same-day myomectomy** | **Same-day removal of another IUD** |  |  |
| **Total N** | 783 | 38 | 40 |  | 861 |
| **Mean (SD)** | | | | | |
| Age | 33.1  (7.4) | 37.2  (5.1) | 33.6  (6.2) | 0.003 | 33.3  (7.3) |
| Uterine bleeding symptom severity Score | 4.9  (4.7) | 8.0  (5.6) | 3.0  (3.6) | <0.001 | 4.9  (4.8) |
| Pelvic pain symptom severity score | 3.3  (3.2) | 3.8  (2.9) | 1.9  (2.8) | 0.013 | 3.3  (3.2) |
| Uterine bulk symptom severity score | 1.6  (1.8) | 3.3  (2.0) | 1.0  (1.6) | <0.001 | 1.7  (1.8) |
|  |  |  |  |  |  |
| **N (%)** | | | | | |
| Race and Ethnicity |  |  |  |  |  |
| White | 455  (58.1) | 11  (29.0) | 24  (60.0) | 0.002 | 490  (56.9) |
| Black | 208  (26.6) | 17  (44.7) | 10  (25.0) | 0.046 | 235  (27.3) |
| Hispanic | 120  (15.3) | 10  (26.3) | <10^a^  - | 0.191 | 136  (15.8) |
| Diagnostic billing codes associated with the LNG-IUD insertion |  |  |  |  |  |
| Abnormal uterine bleeding | 562  (71.8) | 30  (79.0) | 27  (67.5) | 0.516 | 619  (71.9) |
| Gynecologic pain | 331  (42.3) | 13  (34.2) | 14  (35.0) | 0.424 | 358  (41.6) |
| Endometriosis | 120  (15.3) | <10^a^  - | <10^a^  - | 0.161 | 126  (14.6) |
| Fibroids | 83  (10.6) | 36  (94.7) | <10^a^  - | <0.001 | 123  (14.3) |
| Number of above diagnostic categories associated with the LNG-IUD insertion |  |  |  |  |  |
| 1 only | 529  (67.6) | <10^a^  - | 33  (82.5) | <0.001 | 566  (65.7) |
| 2 or more | 254  (32.4) | 34  (89.5) | <10^a^  - | <0.001 | 295  (34.3) |
| Insurance Status |  |  |  |  |  |
| Commercial | 462  (59.0) | 20  (52.6) | 27  (67.5) | 0.401 | 509  (59.1) |
| Medicaid | 150  (19.2) | <10^a^  - | <10^a^  - | 0.767 | 164  (19.1) |
| Uninsured | 142  (18.1) | 10  (26.3) | <10^a^  - | 0.441 | 159  (18.5) |
| Medicare | 29  (3.7) | <10^a^  - | <10^a^  - | 0.224 | 29  (3.4) |
| Parity |  |  |  |  |  |
| Not recorded | 55  (7.0) | <10^a^  - | <10^a^  - | 0.518 | 58  (6.7) |
| Nulliparous | 291  (37.2) | 11  (29.0) | 14  (35.0) | 0.575 | 316  (36.7) |
| Single parity | 119  (15.2) | <10^a^  - | <10^a^  - | 0.633 | 134  (15.6) |
| Multiparous | 318  (40.6) | 19  (50.0) | 16  (40.0) | 0.512 | 353  (41.0) |
| Prior medical treatments^b^ |  |  |  |  |  |
| None | 234  (29.9) | <10^a^  - | 23  (57.5) | <0.001 | 264  (30.7) |
| Single type | 298  (38.1) | 12  (31.6) | 12  (30.0) | 0.443 | 322  (37.4) |
| Multiple types | 251  (32.1) | 19  (50.0) | <10^a^  - | 0.002 | 275  (31.9) |
| Prior surgical procedures^c^ |  |  |  |  |  |
| None | 460  (58.8) | <10^a^  - | 26  (65.0) | <0.001 | 492  (57.1) |
| Single type | 197  (25.2) | 12  (31.6) | <10^a^  - | 0.617 | 218  (25.3) |
| Multiple types | 126  (16.1) | 20  (52.6) | <10^a^  - | <0.001 | 151  (17.5) |
|  |  |  |  |  |  |
| Any prior IUD use |  |  |  |  |  |
| None recorded | 626  (80.0) | 31  (81.6) | <10^a^  - | <0.001 | 660  (76.7) |
| Prior IUD (uncertain type) | 50  (6.4) | <10^a^  - | 15  (37.5) | <0.001 | 68  (7.9) |
| Prior IUD (hormonal) | 107  (13.7) | <10^a^  - | 22  (55.0) | <0.001 | 133  (15.5) |

^a^Cell sizes <10 and their associated percentages are suppressed.

^b^Prior medical treatments included: Depo Provera, GnRH agonists, hormonal contraceptives (oral, patch, or ring), intravenous hormonal injections, hormonal implants, iron supplements, tranexamic acids (Lysteda), or Progestins.

^c^Surgical procedures included: dilation and curettage, endometrial ablation, hysteroscopy, laparoscopy (for gynecologic indication), laparotomy (for gynecologic indication), and myomectomy.

**Supplementary Table S5: Sensitivity analyses with an extra 78 LNG-IUD patients who were excluded from the main analyses**

|  | **Unadjusted model** | **Adjusted model** |
| --- | --- | --- |
| *Age at treatment^a^* | | |
| White (reference) | - | - |
| Black | 1.06  (-0.06 to 2.18) | 0.37  (-0.60 to 1.34) |
| Hispanic | 3.57  (2.19 to 4.94) | 0.50  (-0.79 to 1.79) |
|  |  |  |
| *Uterine bleeding symptom severity score^b^* | | |
| White (reference) | - | - |
| Black | 1.73  (1.50 to 2.00) | 1.36  (1.20 to 1.55) |
| Hispanic | 1.80  (1.51 to 2.15) | 1.20  (1.01 to 1.42) |
|  |  |  |
| *Pelvic pain symptom severity score^b^* | | |
| White (reference) | - | - |
| Black | 0.94  (0.79 to 1.12) | 0.89  (0.75 to 1.05) |
| Hispanic | 0.94  (0.76 to 1.15) | 0.91  (0.73 to 1.13) |
|  |  |  |
| *Uterine bulk symptom severity score^b^* | | |
| White (reference) | - | - |
| Black | 1.59  (1.32 to 1.92) | 1.35  (1.13 to 1.62) |
| Hispanic | 1.62  (1.29 to 2.04) | 1.25  (0.99 to 1.59) |

^a^Estimates are coefficients from linear regression models. Adjusted model includes insurance status, parity, prior treatments, and diagnostic categories.

^b^Estimates represent ratios of scores between exposure groups from negative binomial models. Adjusted model includes age at treatment, insurance status, parity, prior treatments, and diagnostic categories.

**Supplementary Table S6: Sensitivity analyses with 83 LNG-IUD patients who had diagnostic codes for Fibroids**

|  | **Unadjusted model** | **Adjusted model** |
| --- | --- | --- |
| *Age at treatment^a^* | | |
| White (reference) | - | - |
| Black | -0.02  (-2.95 to 2.92) | 0.23  (-2.79 to 3.26) |
| Hispanic | 0.20  (-3.31 to 3.70) | 1.03  (-2.84 to 4.90) |
|  |  |  |
| *Uterine bleeding symptom severity score^b^* | | |
| White (reference) | - | - |
| Black | 1.00  (0.63 to 1.57) | 1.13  (0.80 to 1.59) |
| Hispanic | 1.29  (0.76 to 2.21) | 1.14  (0.72 to 1.81) |
|  |  |  |
| *Pelvic pain symptom severity score^b^* | | |
| White (reference) | - | - |
| Black | 0.92  (0.54 to 1.57) | 0.91  (0.58 to 1.43) |
| Hispanic | 0.91  (0.48 to 1.73) | 0.67  (0.37 to 1.21) |
|  |  |  |
| *Uterine bulk symptom severity score^b^* | | |
| White (reference) | - | - |
| Black | 1.31  (0.91 to 1.89) | 1.31  (0.95 to 1.80) |
| Hispanic | 1.08  (0.69 to 1.69) | 1.02  (0.67 to 1.57) |

^a^Estimates are coefficients from linear regression models. Adjusted model includes insurance status, parity, prior treatments, and diagnostic categories.

^b^Estimates represent ratios of scores between exposure groups from negative binomial models. Adjusted model includes age at treatment, insurance status, parity, prior treatments, and diagnostic codes for endometriosis.

**Supplementary Table S7: Sensitivity analyses with 120 LNG-IUD patients who had diagnostic codes for Endometriosis**

|  | **Unadjusted model** | **Adjusted model** |
| --- | --- | --- |
| *Age at treatment^a^* | | |
| White (reference) | - | - |
| Black | 0.08  (-2.74 to 2.89) | -0.02  (-2.65 to 2.61) |
| Hispanic | 5.84  (1.51 to 10.17) | 1.88  (-2.61 to 6.37) |
|  |  |  |
| *Uterine bleeding symptom severity score^b^* | | |
| White (reference) | - | - |
| Black | 1.69  (1.07 to 2.68) | 1.41  (0.88 to 2.25) |
| Hispanic | 1.89  (0.94 to 3.80) | 1.26  (0.59 to 2.70) |
|  |  |  |
| *Pelvic pain symptom severity score^b^* | | |
| White (reference) | - | - |
| Black | 0.93  (0.73 to 1.18) | 0.89  (0.70 to 1.13) |
| Hispanic | 0.73  (0.49 to 1.09) | 0.83  (0.53 to 1.28) |
|  |  |  |
| *Uterine bulk symptom severity score^b^* | | |
| White (reference) | - | - |
| Black | 1.18  (0.69 to 2.01) | 1.25  (0.72 to 2.16) |
| Hispanic | 2.39  (1.16 to 4.93) | 2.83  (1.25 to 6.38) |

^a^Estimates are coefficients from linear regression models. Adjusted model includes insurance status, parity, prior treatments, and diagnostic categories.

^b^Estimates represent ratios of scores between exposure groups from negative binomial models. Adjusted model includes age at treatment, insurance status, parity, prior treatments, and diagnostic codes for fibroids.
